# Physiotherapists’ perceptions and experiences of home-based rehabilitation in Libya: a qualitative study

**DOI:** 10.11604/pamj.2021.40.256.28841

**Published:** 2021-12-22

**Authors:** Alhadi Mohamed Jahan, Ali Emhemed Rwaiha

**Affiliations:** 1 Physiotherapy Department, College of Medical Technology, Misrata, Libya,; 2 School of Rehabilitation Sciences, University of Ottawa, Ottawa, Canada

**Keywords:** Home-based rehabilitation, experiences, physiotherapy, barriers, qualitative, Libya

## Abstract

**Introduction:**

home-based rehabilitation (HBR) is a rehabilitation model that aims to help people with disabilities to integrate into the community and be independent as much as possible. HBR is a promising alternative to institution-based rehabilitation, in which rehabilitation services are provided at patients' homes. However, challenges and barriers to HBR practice in Libya have never been researched before. This study explores physiotherapists' perceptions of home-based rehabilitation (HBR) in Libya and examines their views and the concerns they face.

**Methods:**

eight physiotherapists (2 females, 6 males) with at least two years of work experience in the Libyan physiotherapy community participated in in-depth semi-structured interviews. The interviews were audio-recorded and transcribed verbatim, and the data were analyzed using framework method.

**Results:**

three themes emerged from the data, namely: i) access problems, including lack of infrastructure; ii) lack of governmental policies, such as the absence of governmental support (e.g., lack of programs and resources); iii) poor awareness and misconception issues, including that of patients and families.

**Conclusion:**

although all the interviewed physiotherapists described HBR as an essential practice in Libya, they expressed concerns about several factors that hinder its development and may influence the quality of interventions provided in the community. Given the fact that this is the first qualitative study in this field in Libya, there is a need for future research to explore HBR from other perspectives, such as those of policymakers, healthcare planners, or patients and their families and/or caregivers.

## Introduction

Home-based rehabilitation (HBR) is an alternative to traditional institution-based rehabilitation that aims to optimize health-related quality of life through delivering rehabilitation services at patients´ homes by rehabilitation professionals such as physiotherapists [1]. There is growing evidence in the literature that supports HBR over other rehabilitation settings [2,3]. Home-based rehabilitation has been documented as an effective intervention for many patient groups, among whom are stroke survivors [4], patients with total knee replacements [5], and patients with neuromuscular conditions [6], to mention a few. In Libya, rehabilitation services are provided in both the public and private sectors. Often, after discharge from hospitals, patients cannot receive rehabilitation services in private facilities, either due to economic reasons or because they are too frail or disabled to travel there regularly [7]. These individuals end up on long waiting lists for public hospitals, and the inherent delays may cause their conditions to worsen. Therefore, HBR is considered an important and relatively cost-effective way of providing rehabilitation services to the public. The healthcare problem in Libya was exacerbated by the war that followed the revolution in 2011 [8]. As a result, the healthcare system, as with many other governmental organizations, collapsed [9]. In early 2012, Libya requested support from the World Health Organization (WHO) to deal with the crippled healthcare system, as the war resulted in large numbers of injured people, often with permanent disabilities as a result of amputations and spinal cord injuries [10]. The current situation in Libya clearly calls for a more practical approach that can provide more efficient rehabilitation services such as HBR.

Despite the benefits and practicality of HBR as it pertains to Libya, there are still gaps in health policy research, and Tashani [11] suggested that Libya needs more research to inform policy developments. Furthermore, little is known about the perceptions of physiotherapists with respect to HBR for people with disabilities in the community. To date, there have been few studies internationally that have examined the views of physiotherapists who manage HBR. Examples of these studies include Carson *et al*. [12] and Hall *et al*. [13]. The interviewees of these studies mentioned several barriers that limit HBR services, including a lack of resources. With regard to Libya, there is a lack of qualitative research in the context of rehabilitation. To the best of our knowledge, this is the first qualitative study focusing specifically on the experiences of physiotherapists who provide HBR in Libya. As rehabilitation services in Libya are provided mainly by physiotherapists, this project was therefore limited to home-based physiotherapy services. Awareness of the perceptions and attitudes of physiotherapists is important, as ingrained beliefs about HBR may restrict its promotion and development. Understanding these views may also improve the therapeutic collaborative relationships between people with disabilities and healthcare providers, in which interns improve the quality of life of home-dwelling patients, relieve the burden on their families, and improve the healthcare system at large. Additionally, this research could provide the foundations necessary to develop and design HBR programs that are efficient and meaningful to the community. To that end, the aim of this research was to explore Libyan physiotherapists´ perceptions, views, and opinions on barriers that potentially influence current HBR practices in Libya.

## Methods

**Research design:** this study was underpinned by phenomenology, as it aimed to achieve an in-depth understanding of reality from physiotherapists´ narratives as related to their experience of HBR services. To that end, qualitative in-depth semi-structured interviews were used.

**Participants and recruitment:** purposeful sampling was used for the identification of the study´s participants [14]. As stated by Cresswell [15], purposive sampling involves identifying and selecting individuals who are knowledgeable or experienced about a given phenomenon. The sample size was determined by data saturation [16]. When we reached the point where no new information were observed, data collection was terminated. However, to ensure the quality of the data collected, when data saturation was observed, two more interviews were conducted in order to make sure that we did not miss any relevant information. Recruitment took place in the city of Misrata (Libya) from August 4^th^ to December 10^th^, 2019. The participants had no previous acquaintance with the interviewer. Variation in years of experience in HBR, gender of participant, and number of geographic locations of work experience were considered in order to obtain diversity in the experiences with HBR. The participants were eligible if they: i) were registered physiotherapists in Misrata city; ii) were able to sign the consent form; iii) were working in HBR practice and had at least two years of work experience in that field. Two years of HBR experience was applied to make sure that potential interviewees had enough knowledge and experience to discuss the concerns and challenges of HBR. The researchers initially approached two rehabilitation institutions and asked them to nominate relevant participants. To screen for the inclusion criteria, the researchers had preliminary face-to-face meetings with nominated participants prior to the interviews to confirm their eligibility and interest. Overall, seventeen physiotherapists were invited to a preliminary discussion; and out of them, eight agreed to participate.

**Data collection:** an interview guide was developed based on a literature review, clinical knowledge, and research experience see Table 1. The interview guide was piloted with professionals who were familiar with the topic, and minor modifications were made for simplicity and clarity. The researchers contacted potential participants via the phone and invited them to a face-to-face meeting. The meetings were scheduled based on the participants´ preferences, and were held in a private office in the participants´ workplaces. In the meeting, the study's purpose was explained, and the consent form was provided. The participants returned their signed consent forms before their appointments for the interviews. Semi-structured, in-depth telephone interviews were conducted at times that were convenient for the participants. With the participants´ permission, all interviews were audio-recorded and lasted between 30 to 45 minutes. The interviews, were in the Arabic language, and the quotations were translated to English by professional translators who were not in the research team by using the forward-backward method [17].

**Table 1:** interview guide

Topic area	Question examples
Introduction	Self-introduction
	Could you please tell me about your experience in physiotherapy?
Context	Could you tell me about what you know about home-based rehabilitation?
	What are your thoughts about working in home-based rehabilitation? Is it effective? Is it feasible?
	Would you be able to describe your experience working in home-based rehabilitation in Libya?
Thoughts	Tell me about your experience working in a home-based rehabilitation program; what were the things about the program that worked well?
	What were the things about the program that did not work well?
	How do you feel about the challenges/barriers you face in applying home-based rehabilitation? What could be done to improve the program? Any thoughts
The future	What would you change about the home-based rehabilitation program?
	Would you like to add anything else?
	Let each participant know that you would be asking them questions about their experience in home-based rehabilitation in Libya
	Remind each participant that you would be recording their answers so that we could analyze them later for research purposes only
	Remind each participant that their participation in the interview was entirely voluntary and would have no effect on their work hours in this centre
	Remind each participant that they had the right to stop the interview or to refuse to answer any question that they did not want to answer
	Remind each participant that if they felt uncomfortable, they had the right to reschedule the telephone interview to a later time

**Data analysis:** the phone interviews were audio-recorded and transcribed verbatim by a professional transcriber. The transcripts were read several times by (AJ and AR) independently to get an overall understanding of meanings of the content. During this process, if clarification were needed, the participants were contacted, and the transcripts amended. The data were analyzed using the Framework Method [18], which involves five iterative steps: familiarization with the primary data, coding, identifying a thematic conceptual framework, applying the analytical framework, and charting data into the framework matrix [18,19]. The data analysis method was selected based on three main criteria: the research question, the nature of the data, and the pragmatics of working together in a multidisciplinary research team. The Framework Method of analysis was chosen because it meets the above criteria [20].

**Rigor:** to establish the trustworthiness of our data, we followed the recommendations by Shenton [21]. Credibility was achieved by conducting semi-structured in-depth interviews with an experienced qualitative interviewer, and the collected data were validated by employing a peer-debriefing technique [22]. Also, data analysis of the transcripts was done independently by the two authors of this study (Alhadi Mohamed Jahan and Ali Emhemed Rwaiha). The findings were then discussed by the research team and any disagreements were solved using the consensus technique [23]. Furthermore, the transcriptions were sent to the participants for them to confirm and to suggest changes in case of any misunderstandings. To address transferability, we relied on the variability of interviewees´ characteristics, including their work experience, as well as the rich variety of thoughts and quotations collected in the interviews. Moreover, the researchers who conducted this study came from the same community and work environment as those of the participants; this made the process of analyzing and interpreting the data more straightforward. Finally, the researchers attempted to provide sufficient details about the research methodology, context, and data collection in this report; this would enhance the reproducibility and the transferability of this study [24].

**Ethics, consent, and permissions:** ethical and research governance approvals for this project were obtained from the College of Medical Technology, Al-Tadamon Rehabilitation Centre and Alpha Medical Centre in Misrata, Libya Ethics certificate# EXT-198-2019. The objectives of the study and the voluntary nature of participation were discussed with the participants during face-to-face individual meetings with the researchers. The participants provided the signed consent form, including consent for being audio-recorded, before scheduling the telephone interviews. To ensure confidentiality, codes were used (e.g., PT3, PT5 etc.) instead of the participants´ names. We also did not collect any identifying information such as age or city of origin due to the limited number of physiotherapists who are involved in HBR in Misrata region.

## Results

The sample consisted of eight physiotherapists (2 females, 6 males) with experience working with people with disabilities in HBR programs ranging from 4 to 10 years see Table 2. Three main aspects of the challenges facing the physiotherapists in HBR were described from the data and grouped according to three themes: access problems, lack of governmental policies, and poor awareness and misconception issues. These three themes and the corresponding subthemes are presented in the following section.

**Table 2:** participant characteristics

Code	Gender	Qualification	Experience
PT1	Male	Bachelor's degree	5 years
PT2	Male	Bachelor's degree	5 years
PT3	Male	Bachelor's degree	10 years
PT4	Male	Bachelor's degree	4 years
PT5	Male	Bachelor's degree	10 years
PT6	Male	Bachelor's degree	4 years
PT7	Female	Bachelor's degree	5 years
PT8	Female	Bachelor's degree	5 years

### Access problems

**Patients´ capability:** patients´ capability was the most common subtheme reported by the participants. The participants agreed that a patient's physical capacity to do tasks as recommended by their physiotherapist is considered an essential factor that controls the delivery of HBR. *PT6: “Most patients with mobility limitations need to use a wheelchair or other device to help them to move, so if they do not have any assistive devices at home, physiotherapy will not be as effective as it is supposed to be, especially in the first few weeks after injury.”* Any patient who is experiencing physical impairment, is bedridden, or has limited mobility is a potential candidate for HBR. However, patients who are cognitively or intellectually challenged or are medically fragile due to a chronic condition which results in a prolonged dependency on medical care for which daily skilled nursing intervention is medically necessary, renders HBR services next to impossible. *PT8: “Patients with complicated medical conditions cannot have HBR sessions at all. This also applies to elders with cognitive problems who require a caregiver available at all times to support the physiotherapist during the treatment session.”*

**Lack of infrastructure:** some participants thought that a lack of public transport was a barrier to HBR applications, as this restricts the ability of physiotherapists to be involved in HBR. Also, poor transportation further limits the ability of physiotherapists of low socio-economic levels who do not own vehicles to access patients´ homes to provide treatment. *PT2: “you know, in Libya; we do not have public transports like buses. Therefore, you have to rely on your private car or a taxi, which are not always available.”* Furthermore, participants talked about other barriers like poor road conditions, especially in the winter months. For example, in rural areas, the roads and infrastructure are deficient or lacking; therefore, lack of access to patients who live in such areas is an obstacle to the implementation of HBR. *PT3: “The unpaved roads prevent physiotherapists from accessing their patients at home easily you know, in the winter months, roads are literally blocked due to accumulated rainwater, maybe because of poor drainage systems, I really do not know.”*

### Lack of governmental policies

**Absence of government support:** from the participants´ point of view, the absence of government support for HBR programs has an adverse effect on service delivery. Since patients often require equipment, the absence of funds from the government affects the progress of HBR. *PT4: “The government should support the patients and provide them with assistive devices or other expensive in-home devices that facilitate treatment sessions... ”*

**Poor medical management:** limited documentation of patient information and poor communication between healthcare institutions was a significant barrier mentioned by all the interviewees. From the participants´ point of view, physiotherapists need to get access to a patient´s information beforehand in order to get a good picture of their condition. Equally important, awareness of other environmental factors is essential for planning a treatment program that accommodates important ethical, psychological, and social considerations. *PT5: “The policymakers in the healthcare system need to develop programs for better documentation and communication between healthcare providers in order to help physiotherapist to provide HBR services efficiently...”*

**Lack of collaboration and teamwork:** a few interviewees mentioned the role of collaboration with other healthcare professionals, as some patients need more than just physiotherapy. For example, patients may sometimes be frail seniors who need neurologists, psychologists, or social workers in addition to physiotherapists or occupational therapists. Therefore, working as a team during the physiotherapy process could positively influence the rehabilitation outcomes. *PT1: “ ...legislation by the Ministry of Health and the Ministry of Social Works for implementing HBR lacks important details about teamwork...”* Moreover, participants stressed the importance of multidisciplinary teams in providing care for those living at home: *PT7 : “ ...Home-based rehabilitation programs should include a team of healthcare professionals such as physicians, psychologists, and occupational therapists in order to achieve better outcomes...”*

**Poor awareness and misconception issues:** poor awareness of HBR and how it differs from institution-based rehabilitation can cause several problems. According to most of the participants, awareness about HBR services plays an essential role in the effective and smooth delivery of such services. The participants highlighted two components of poor awareness and misconception that should be considered: patients´ and family members´. Most of the participants agreed that a lack of patient and family knowledge regarding home visits provided by physiotherapists could interrupt the treatment plan, as many questions and misconceptions may arise after the beginning of the program. In other words, the greater the awareness, the better the delivery of HBR services.

**Patients´ poor awareness and misconceptions:** a few participants voiced that patients often thought that having treatment sessions at their home was not appropriate for them. *PT3: “ ...some patients think that physiotherapy Also, when patients believe that their disability is a result of the aging process and that physiotherapists cannot make it any better, their misconception complicates the treatment plan. PT6: “...elderly patients believe that they experience mobility limitations because they are old and therefore, physiotherapy will not help them. ”Furthermore, patients sometimes believed that their homes were very personal spaces, and they did not want any “strangers” there. PT4: “...some patients say that their home is a private place and they refuse any strangers wanting to come into their home, and if you did, they look at you as being unwelcome it the situation is uncomfortable for everyone. ”Moreover, participants repeatedly mentioned that patients often request physiotherapists PT1: “Some patients stated that they would prefer to see a physiotherapist they were already familiar with and they trusted to deliver HBR.”* One participant highlighted the importance of patient education in promoting awareness and perceptions about HBR services. *PT3: “I have been working for years in home-based rehabilitation, and none of my patients have ever had any educational training about their condition or about HBR.”*

**Family members´ poor awareness and misconceptions:** most home-based patients receive some level of care from their family members, but this informal care can be significantly interrupted in scope, duration, and intensity due to misperceptions about HBR. Often, informal caregivers think that HBR can be substituted with care provided by family members. *PT2: “Family members sometimes stop providing support for patients when HBR starts they think that physiotherapy visits are enough to cure their loved ones.”* Likewise, participant PT1 said: *“There is a general belief among family members that physiotherapists are healthcare professionals who can treat the patients alone, without any help from the families, which is wrong we physiotherapists need to work together with family members to achieve meaningful outcomes.”*

## Discussion

This research aimed to explore Libyan physiotherapists´ experiences and views about barriers to HBR services and to discuss how these insights may influence current HBR practices in Libya. The views of the interviewees were grouped into three main themes and seven subthemes. These views may help clinicians and policymakers in Libya and beyond to implement effective strategies that accommodate essential evidence-based practices. While many of these practices have been reported in the literature, our interviewees highlighted novel insights specific to developing countries like Libya. Evidently, this qualitative study is the first of its kind done in Libya that addresses the challenges and barriers in HBR. Our interviewees agreed that HBR is of utmost importance for the health care system in Libya today, as a large number of people have need for it, especially due to the ongoing war that started in 2011 following the revolution [8]. The challenges in the healthcare system are exacerbated by the closures of healthcare institutions due to violent attacks or due to lack of staff and equipment, as documented by the World Health Organization [25]. Therefore, as expected, the findings from the current study highlight several challenges that make HBR delivery in Libya complicated at this time. All the participants agreed that accessibility and patients' capabilities are the most critical factors influencing HBR delivery. By “patients' capabilities”, we mean the physical and cognitive capabilities of home-dwelling patients to fulfil and adhere to rehabilitation treatment plans. As our interviewees recognized, patients´ physical and cognitive abilities determine the success of HBR, because patients with complex medical conditions, such as stroke survivors, those with head and spinal cord injuries, or with other neurological conditions may find it hard to follow treatment plans effectively. Patients' cognitive and physical functioning may determine the success or failure of HBR; for example, patients with communication problems, multimorbidity, or frailness may pose challenges to physiotherapists when treating these populations [26,27]. In line with this theme, a recent qualitative study by Hall *et al*. (2017) suggested similar challenges for physiotherapists when treating patients with dementia [13], and similar experiences have been reported by physiotherapists in patients with neurological and palliative problems [12].

In the context of Libya, home-dwelling patients most likely have advanced medical conditions (e.g. cancer) or permanent disabilities (e.g., spinal cord injuries). Given the fact that there are no health insurance plans that cover important equipment, such as mobility assistive devices for instance, patients may end up bedridden for months and even years, and physiotherapists face multiple problems related to decreased mobility, which of course requires a lot of work and effort. Adding to the problem, patients sometimes experience cognitive or behavioral problems in addition to physical impairment which further hinder the provision of HBR services. Consistent with our findings, research has documented that physical and cognitive impairments make healthcare services for patients with such impairments more complicated [28,29]. Patients with physical and cognitive impairments pose extra challenges for their physiotherapists because they require more supervision, often experience behavioral problems, are unlikely to express gratitude, and are most likely to experience stress and depression [30]. Physical and cognitive impairments can also put extra pressure on physiotherapists to ensure their patients´ safety. Mental burdens are relatively high in the Libyan community as reported in literature [31]. These burdens pose several risks to patient safety in the home setting, such as environmental hazards, risk of falling, and patient education problems, to mention a few. Evidently there is a large research gap in Libya, as we do not know the prevalence of physical and mental burdens among patients who receive healthcare services at their homes, and we encourage further research in this regard. Therefore, assessing safety risks as well as access options, and determining clear inclusion and exclusion criteria when moving to HBR is warranted.

In order to meet the HBR goal, it was essential to first understand the barriers and challenges that face physiotherapists in Libya with regard to HBR delivery. Conceptualizing the HBR programs in Libya in a holistic way (i.e. thinking outside the box) might be necessary at this challenging time as the healthcare system struggles to address the basic medical needs of the community [32]. Therefore, it is imperative to plan HBR services as an “integrated community program” that benefits from a wide array of available community resources [1,33,34]. Thus, it is essential to involve community partners, such as non-governmental organizations, in HBR service delivery. This suggestion was mentioned by six out of the eight interviewees as a potential facilitator for HBR in Libya in the coming months or years. Another level of cooperation that was mentioned by our interviewees is networking with other healthcare professionals at the intermediate level. At this level, good communication with healthcare professionals in both private and public healthcare institutions could benefit not only the quality of service delivery, but also the development of HBR programs. Based on the participants´ views, this can be achieved by offering referral services when needed and providing training and technical supervision to rehabilitation personnel. All the aforementioned dimensions of professional networking have been discussed in the literature [33,35,36]. This view was further supported by a recent qualitative study in the United Kingdom. In that study, the researchers used a qualitative interview process that included eleven physiotherapists involved in delivering HBR in palliative care, and highlighted lack of cooperation with other healthcare professionals as a barrier to the provision of HBR to palliative patients [12].

Poor awareness and misconception is another theme that emerged from the interviews with our participants. The interviewees agreed that the levels of awareness and attitudes among patients and their families play an instrumental role in HBR delivery. Some interviewees also connected levels of family awareness with treatment outcomes, as they noted better treatment outcomes when the patient and their family were aware of HBR´s goals and services. However, the interviewees did not hide their concerns about the difficulties in the implementation of the HBR model to urban regions due to misconceptions over the definition of HBR among the diverse cultural and ethnic groups in Libya. A few limitations of this study should be noted. First, the researchers used phone interviews to conduct the study, making it impossible to record non-verbal signals, which might affect the quality of the collected data. However, to ensure the accuracy of the collected data, transcriptions were sent to participants to confirm the transcriptions´ content. Second, all the participants were physiotherapists, so the findings of the current study stemmed solely from their points of view without considering patients´ or families´ opinions, which might affect the generalizability of the results. Also, the gender distribution of the interviewees was not equal, as we interviewed two females and six males. This gender imbalance is due to the fact that the majority of physiotherapists who provide HBR in Libya are males. Only a few females provide HBR services, and most of the females who were invited declined to participate in this study.

## Conclusion

Overall, this study provides the first qualitative attempt to explore physiotherapists’ experience with HBR from the point of view of the current challenges in Libya’s healthcare system. Moreover, the aim of this study was to present a useful starting point for the implementation and sustainable management of HBR services by approaching the process from the reality of practice and in relation to the available rehabilitation services. The interviews revealed challenges related to patients’ capabilities as well as access, government policies, and public awareness about the HBR model. By addressing these challenges, we can ensure that HBR services are delivered in a way that contributes to the best possible outcomes. Finally, it is worth noting that the current study explored the experiences of physiotherapists delivering care to a variety of patient populations, and therefore it is imperative to focus future research on addressing HBR challenges from the perspectives of people receiving these services as well as their family members and or caregivers.

### What is known about this topic

- *Home-based rehabilitation is an effective rehabilitation model that can relieve the stress on the healthcare system by providing services at patients’ homes;*
- *Home-based rehabilitation can benefit a large population, including stroke survivors, patients with spinal cord injuries, and the elderly, to mention a few;*
- *Limited resources is an acknowledged barrier to its application in western societies*.

### What this study adds

- *Public's poor awareness and misconception about the HBR model is a big challenge in Libya;*
- *Improved documentations and communications between healthcare institutions can enhance the applicability of HBR;*
- *Future research should focus on the community-dwelling patients as data on the prevalence of medical, psychological, and social burdens among such populations in Libya are not available at this time*.
